# The Mechanism of Effect of Flux Bands on The Arc Behavior in Flux Bands Constricting Arc Welding Process

**DOI:** 10.3390/ma13071652

**Published:** 2020-04-02

**Authors:** Lei Wang, Jisen Qiao, Jianhong Chen

**Affiliations:** 1 School of Materials Science and Engineering, Lanzhou University of Technology, Lanzhou 730050, China; aswl8888@163.com (L.W.); zchen@lut.cn (J.C.); 2 State Key Laboratory of Advanced Processing and Recycling of Nonferrous Metals, Lanzhou University of Technology, Lanzhou 730050, China

**Keywords:** flux bands constricting arc, ultra-narrow gap welding, arc behavior, arc stability, T-joint, weld formation

## Abstract

A new welding method, flux bands constricting arc (FBCA) welding, is proposed to compensate for the shortage of insufficient weld width of laser welding T-joints in high steel sandwich panels. The arc behavior (arc burning position, arc shape, arc heat, and arc stability) before and after sticking the flux bands (GMAW and FBCA welding) to the ultra-narrow gap groove was tested. Results indicate that flux bands have solid-wall constricting effect (SWCE) and thermo-compression effect (TCE) on the arc and self-producing slag and gas function in FBCA welding. In ultra-narrow gap groove, the arc burning position climbing up phenomenon (APCP) occurs without flux bands. The SWCE of flux bands on the arc effectively suppresses the APCP because of the insulation of flux bands. In the FBCA welding process, the effective heating area of the arc is increased by at least 5 mm^2^ compared with that in GMAW. When the groove gap decreases, flux bands not only compress the arc from an inverted bell shape to a rectangular shape, but also make the 660 °C isotherm on the core-plate to increase from 3 mm to 8 mm. In the end, the proportion of unstable arc burning time is reduced by 86.85%, the fluctuation of arc voltage and welding current are also significantly reduced by the flux bands because of their SWCE on the arc.

## 1. Introduction

Today, energy saving and the development of environment-friendly industry are the most important topics, especially lightweight high-speed vehicles have been the focus in the field of energy saving and environmental protection. As a new lightweight structure, high-strength steel sandwich panel (structures with a web-core and two face-plates) [1] provides an effective solution for lightweight high-speed vehicles because of the great impact resistance and high specific strength [2,3,4]. But the welding of the smallest repeating element (T-joint) in this periodic closed structure is one of the key problems. Nowadays, the laser lap deep welding method is mainly used to weld the high-strength steel sandwich panel around the world. However, because of the limitation of characteristics of laser heat source and power, it is hard to obtain sufficient width in the T-joint. Moreover, the weld width decreases with the increase in thickness of the face-plate [5]. There are two obvious gaps between the face-plate and the core-plate [6,7] in the laser welding of the T-joint in the sandwich panel. Jiang indicated that T-joint is the weakest element in the service life of the laser-welded sandwich panel because of the gaps between the face-plate and core-plate [8]. Romanoff [9] and Jelovica [10] pointed out that the insufficient width of the joint significantly affected the service strength of the sandwich panel. In order to compensate for the defect of insufficient weld width of T-joint of laser welding high-strength steel sandwich panel, flux bands constricting arc (FBCA) welding method [11] is proposed to manufacture high-strength steel sandwich panel.

Flux bands constricting arc theory is primitively proposed by Zhu to be used in ultra-narrow gap welding [12]. For thick plate welding, compared with the gas metal arc welding (GMAW) method, the ultra-narrow gap welding has the advantages of no groove, lower heat input, lower equipment investment, less heat affected zone (HAZ) softening [13,14]. However, the arc behavior in ultra-narrow gap is significantly different from the normal GMAW welding. Zhang pointed out that the arc burning position in narrow gap groove will be climbing up along the sidewalls [15]. Meng studied the effects of different groove gaps on arc behavior in laser-arc hybrid welding, pointing out that narrow gap have space constraint effect (SCE) on the arc, which increases the energy transfer efficiency by concentrating arc energy [16]. Several studies indicated that narrow gap significantly affects the arc behavior and metal transfer modes, and also pointed out ultimate ultra-narrow gap is bad for weld stability and weld formation without taking extra actions [17,18,19]. Kang [20] and Xu [21] developed a magnetron swing arc system to control the arc burning position in ultra-narrow gap. Sun [22] further proposed a magnetic arc oscillation system for tungsten inert gas (TIG) narrow gap welding in order to prevent insufficient sidewall fusion and improve efficiency and quality for thick component welding. Cai [23] subsequently used the simulation method to study the characteristics of the welding heat source for tandem narrow-gap gas metal arc welding with different ternary shielding gas (Ar-CO_2_-He) compositions. Guo [24] proposed a rotating arc ultra-narrow gap welding system to alter the staying time of arc on the sidewalls and the arc attachment point for obtaining sound weld formation. Wang [25] systematically studied the oscillating arc narrow gap all-position welding system to optimize the weld bead geometry and estimate weld defects generated by a narrow gap. Zhang [26] put forward laser-arc hybrid method for narrow gap welding, and the microstructure and mechanical properties of the joints were studied. Ribeiro [27] presented a cold wire GMAW method for narrow gap welding of high strength low alloy steel. However, arc behavior and metal transfer characteristics in FBCA welding method are rarely studied. The arc behavior in the ultra-narrow gap and the mechanism of the effect of flux bands on welding arc are still not clear. Consequently, it is necessary to systematically study the effect of flux bands on arc behavior in the ultra-narrow gap, for obtaining stable FBCA welding process.

In this paper, a high-speed camera system is developed to capture the arc behavior, an arc contour extraction method is carried out to study the arc burning position and arc shape, an infrared temperature testing system is used to observe the arc heat, and a real-time electric signal acquisition system is used to analyze the arc stability of the GMAW and FBCA in ultra-narrow gap groove with different groove gaps. The results of this study will provide theoretical basis and process support for FBCA welding high-strength steel sandwich panels.

## 2. Experimental Setup and Procedures

**Figure 1:**
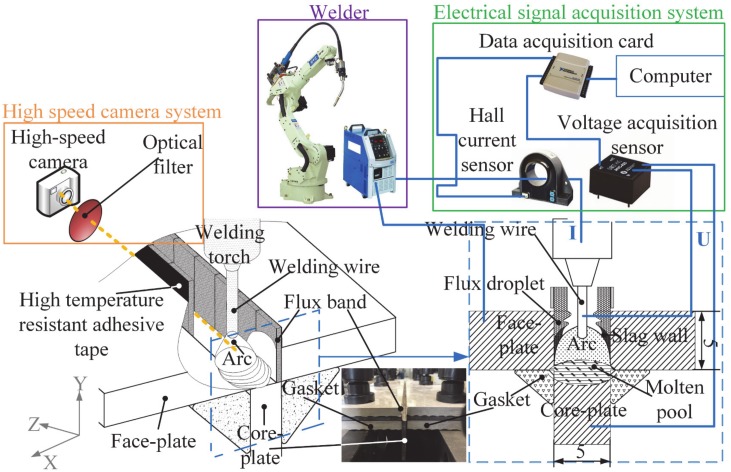
Schematic diagram of flux bands constricting arc (FBCA) welding.

FBCA welding system is shown in Figure 1, which involves sticking the flux bands onto the inside sidewalls of the groove, sticking the welding wire into the square groove, and striking the arc on the core-plate. The welding system was set up with OTC DM500 CO_2_/MAG welding power and the OTC FD-V6 welding robot. BS960 high-strength steel, commonly used for marine sandwich panels, is used in the experiment as the base material of samples. The welding wire is ø1.2-mm high-strength steel welding wire ER120S, and the wire extension length is set to 12 mm. The shielding gas in GMAW welding is: 80% Ar + 20% CO_2_, the gas flow rate is 15 min^−1^. Although it is not necessary to use the shielding gas in the FBCA welding process. The three experimental cases performed are as shown in Table 1. The first and second cases aim at determining the best process parameters (welding current and arc voltage) in the FBCA welding process. Whereas the third case of the experiment focused on studying the arc behaviors in GMAW and FBCA welding process under different groove gaps.

A high-speed camera system is used to capture the arc behavior during welding. The high-speed camera system is mainly composed of horizontal movement welding platform, IDT Y3 high-speed camera, 660 nm interference filter, data acquisition card, and a computer. The image acquisition speed is 3000 frames/s. At the same time, a real-time electrical signal acquisition system is established for the accurate determination of arc stability in the welding process. The system consists of a Hall current sensor (LT 508-S6), a Hall voltage sensor (LV 25-P), a data acquisition card (USB 6009) and a computer. The computer carried out the real-time processing of the signal acquisition thorough the procedures written in LABVIEW.

**Figure 2:**
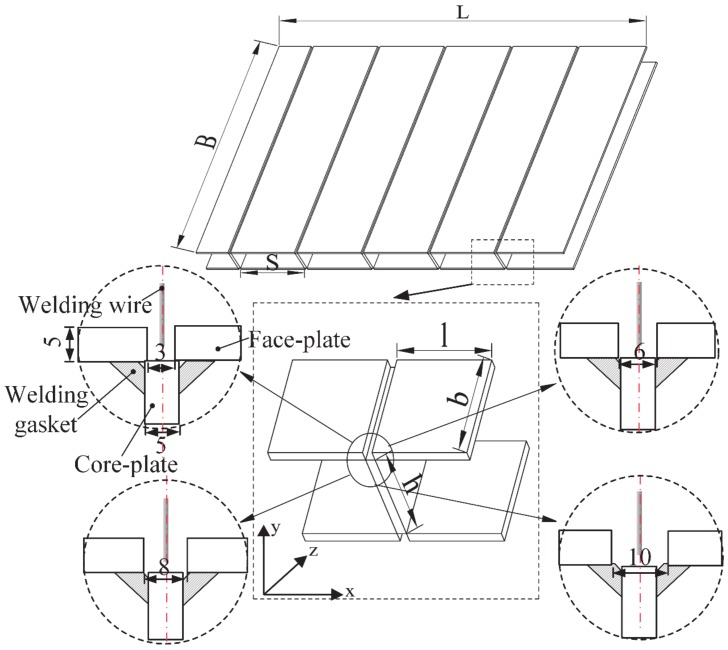
Dimensions of T-joint grooves.

**Figure 3:**
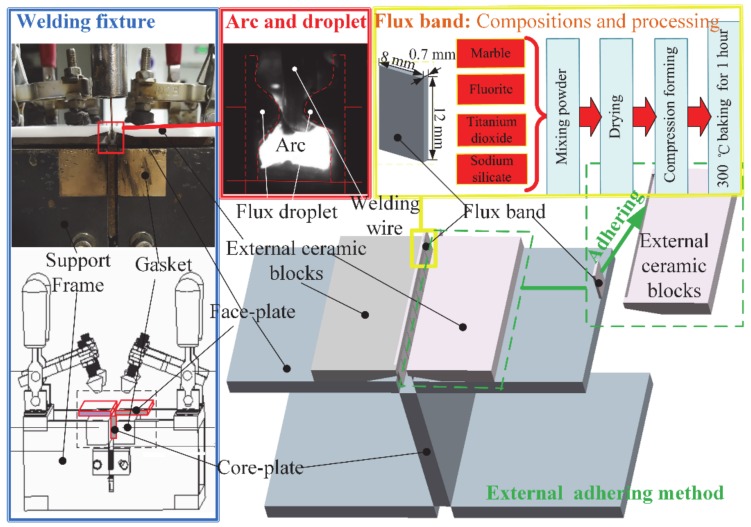
Flux bands and outside adhering method of FBCA welding.

Before welding, the end-sides of the samples were cleaned by mechanical grinding, and samples were assembled by tack welding. The sample sizes are 50 mm × 50 mm × 150 mm, the thickness of the both face-plate and core-plate is 5 mm. The groove styles are designed as shown in Figure 2; the groove gaps are 3 mm, 6 mm, 8 mm, and 10 mm respectively. The external adhering method (adhere flux bands on end-side of external ceramic blocks by high temperature resistant tapes, and place the ceramic blocks on the face-plate side by side) was used to ensure the flux bands are stable in groove as shown in the Figure 3. Single flux band has a length of 12 mm, a width of 10 mm, and a thickness of 0.7 mm. The flux band in this experiment is formed by pressing the powder mixture of marble, fluorite, and titanium dioxide. The detailed compositions of flux band used in this experiment is shown in Table 2.

## 3. Results and Discussion

### 3.1. Flux Bands States in High Temperature

**Figure 4:**
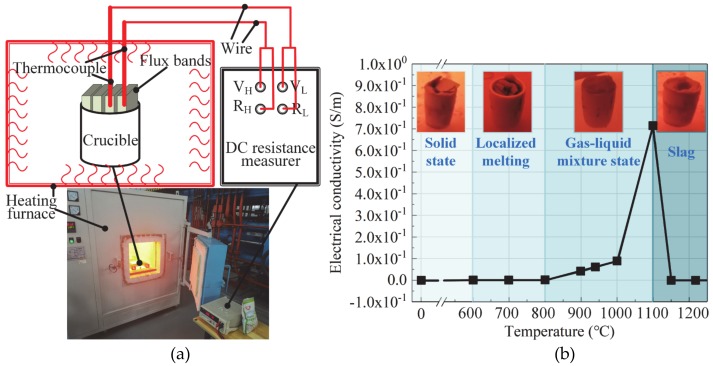
Schematic of (**a**) test experiment and (**b**) results of electrical conductivity of flux bands.

A test experiment of flux bands states at different temperature is proposed, as shown in Figure 4. The experimental results show that the flux bands localized melting starts at 600 °C, then a gas–liquid mixture state occurs at 800 °C. Regardless of the states, the electrical conductivity of flux bands is less than 10^5^ S/m (≥10^5^ S/m is considered as a conductor). Therefore, the flux droplets are regarded as nonconductive. When the flux bands are pasted on both end-sides of face-plates, the non-conductive flux bands will have a solid-wall constricting effect (SWCE) on the arc. On the one hand, it will compress the arc shape, on the other hand, it will prevent the electric arc from conducting with the side-walls, and keep the stable burning of the arc in the core-plate.

**Figure 5:**
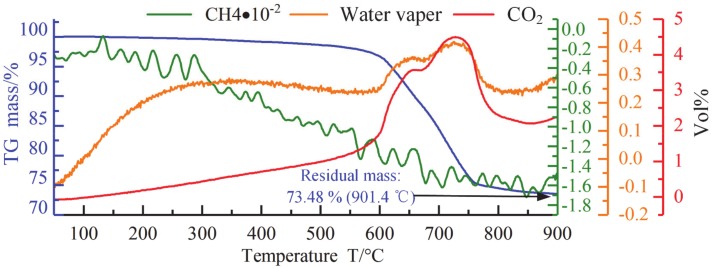
TG-IR analysis of flux bands.

Figure 5 shows the results of TG-IR combined analysis on a single flux band. It can be seen from the figure that, as mentioned above, the flux bands decompose when heated at 600–800 °C and enters a gas–liquid mixture state likes bubble “cheese,” which produces water vapor and CO_2_ as the main gas. CO_2_ content is much higher than other gases, thus becoming the main protective gas atmosphere in the welding process. Finally, the flux band is transformed into welding slag. According to the TG experiment, comparing the mass of flux band before and after fusion, the mass of welding slag is 73.48% of that of the original flux band. As part of the heat of the arc is used to melt the flux bands, the flux bands will decompose and take away part of the heat of the arc. Therefore, in addition to the above-mentioned SWCE of flux bands on the arc, it will also have a thermo-compression effect (TCE) on the arc.

It can be seen from the above analysis that welding slag and CO_2_ gas are produced in FBCA welding, which can protect the welding process with the function of self-making slag and gas. Further, from the main components of the flux bands shown in Table 2, it can be seen that the main chemical reaction formulas of the flux bands in the welding process are as follows.

CaCO_3_ = CaO + CO_2_ (545–910 °C) (1)

Na_2_O•nSiO_2_ + mH_2_O = 2NaOH + nSiO_2_(m–1) H_2_O (2)

2NaOH + CaF_2_ = 2NaF + Ca(OH)_2_ (3)

CaF_2_ + H_2_O = CaO + 2HF (4)

CaF_2_ + 2H = Ca + 2HF (5)

NaF + HF = NaHF_2_ (6)

These reaction formulas indicate that the compositions of flux bands are similar to that of the low hydrogen electrodes, and the flux bands form the CaO-CaF_2_ slag system. The marble consists of CaCO_3_. At temperature of around 600 °C, CaCO_3_ decomposes and produces CO_2_ gas and CaO slag. CO_2_ gas plays the role of shielding gas in FBCA welding process, therefore the effect of gas atmosphere on arc behavior in FBCA welding is similar to that in MAG. CaO transfers to molten pool by combining with the droplets in the form of slag. CaO is not only beneficial for the dephosphorization and desulfurization ability of slag, but also improves the resistance to hot cracking. The component of fluorite is CaF_2_. NaF and HF are produced by the reaction between CaF_2_ and sodium silicate, water vapor and hydrogen in the air. NaF then reacts with HF to produce NaHF_2_. Dehydrogenation is achieved by the above reaction process. Sodium silicate (Na_2_O•nSiO_2_) not only plays the role of binding the components, but also reacts with CaF_2_ in the fluorite and improves the slag performance.

### 3.2. Determination of the Best Process Parameters (Welding Current and Arc Voltage) in FBCA Welding

**Figure 6:**
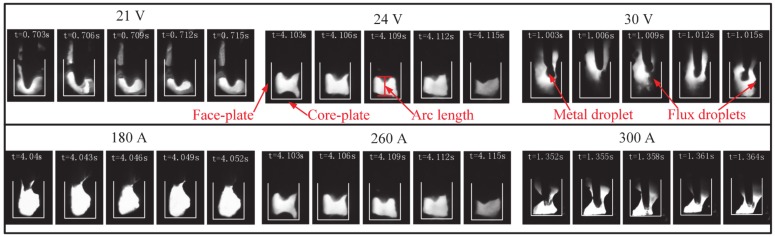
The arc behavior under different arc voltages and welding currents.

The welding process parameters (welding current and arc voltage) are the important factors of ensuring stable FBCA welding process. According to the experimental parameters of case 1 and case 2 as shown in Table 1, the arc behaviors under different welding currents and arc voltages are shown in Figure 6. It can be seen that when the voltage is 21 V, the arc length is shorter than the groove depth, and the arc burns at the root of the groove. When the arc voltage is increased to 24 V, the arc length increases with the increase of voltage. The arc becomes square shaped, and the height of the arc heating the side-walls is almost equal to the groove depth, so the arc effectively heats the core-plate and the side-walls of face-plates at the same time. With a further increase in the arc voltage to 30 V, the larger diameter of the arc cathode spot leads to the faster burning of the flux bands, hence the SWCE of the flux bands on the arc weakens. So, the arc burns above the side-wall of the face-plate, making it difficult to effectively heat the root of the groove. At the same time, comparing the arc behaviors at 180 A, 260 A, and 300 A, it can be seen that with the increase of welding current, the whole heat source moves down. When the welding current is 180 A, the lower arc heat results in the insufficient melting of the flux bands, and the arc is under the strong SWCE of flux bands. The arc is squeezed to a rectangular shape by flux bands, which is bad for the fusion of the side-walls and the core-plate. On the contrary, when the welding current increases to 300 A, the height of the arc heating the side-walls is lower than the groove depth because of the focus heating of arc on the root of the groove, which is not beneficial to the side-walls fusion. When welding current is around 260 A, the arc can effectively heat the face-plates and core-plate. Therefore, in order to further study the effect of flux bands on arc behavior under different groove gaps, welding parameters are selected as: welding current: 260 A, arc voltage: 24 V as shown in case 3 of Table 1.

### 3.3. Effect of Flux Bands on Arc Burning Position

#### 3.3.1. Arc Burning Position Climbing up Phenomenon (APCP)

**Figure 7:**
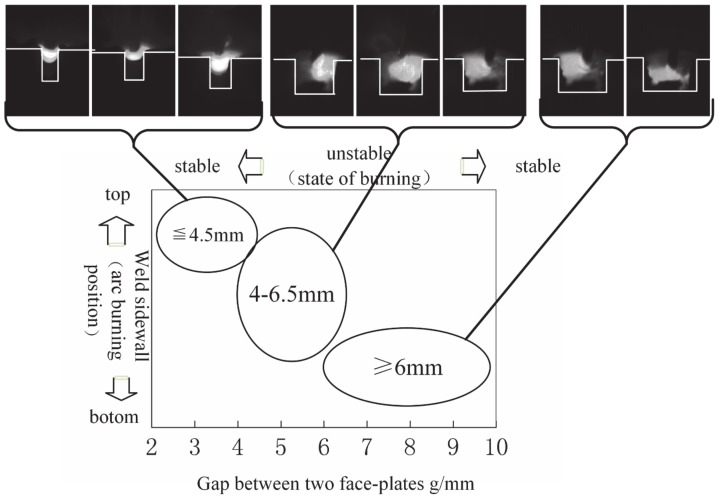
Relationship between weld groove gap and arc burning position.

In the ultra-narrow gap T-joint groove (<10 mm), the GMAW welding arc burning states and positions with various groove gaps are shown in Figure 7. Figure 6 depicts three apparent points: (1) With the decrease of groove gap, the arc attachment point moves from the bottom to the sidewalls of the face-plate and finally to the top of the face-plate; (2) the arc conductive path is always altered in the ultra-narrow gap as the arc attachment point selects the nearest conductive position of the groove; (3) the arc extinction rate increases as the arc conductive path changes in the ultra-narrow gap, which destroys the arc stability.

When groove gap is larger than 6.5 mm, the arc burns on the one side root of the groove. The arc burning position continuously changes from one side root to another as the distance fluctuates between the wire tip and the sidewalls because of the welding wire movement. The fusion metal and molten pool is asymmetric. When the gap decreases to 4–6.5 mm, the arc burning position is on the sidewalls of groove. It is observed that, as the droplet grows, the arc anode spot moves from wire tip to the bottom of the droplet. At the same time, the arc attachment point rotates on the sidewalls as the droplet rotates in the ultra-narrow gap. Ultra-narrow gap constricts the droplet growth causing the droplet to frequently come in contact with the sidewall, generating high arc extinction rate that damages the arc stability. When groove gap decreases to less than 4 mm, no matter where the arc strikes on, the arc burning position will move to the top of the groove along the side wall. The arc length is too short, and stably burns on the upper side of the face-plate. 

**Figure 8:**
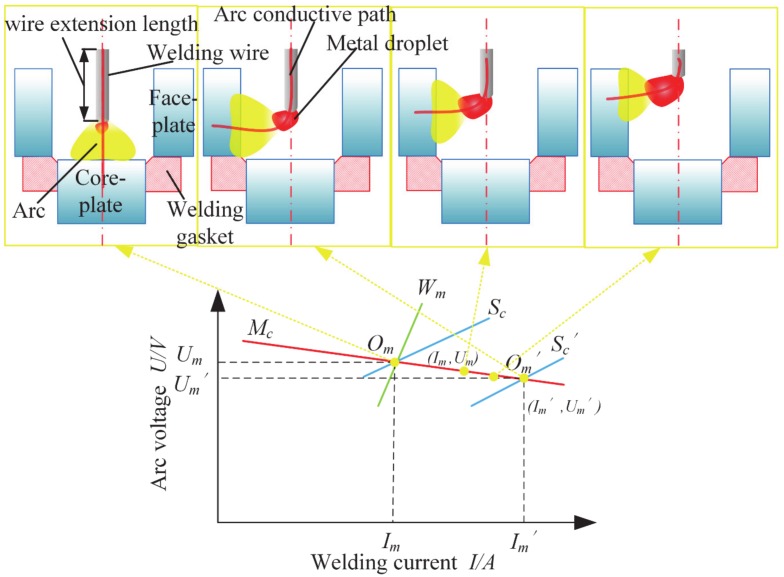
The arc burning position climbing up phenomenon (APCP) process in ultra-narrow gap groove. *M_c_* is the curve of external characteristic of the welding machine, *W_m_* is the curve showing that the wire melting rate equals to the wire feeding speed, *S_c_* is the curve of arc static characteristic, *S_c_^´^* is the curve of short arc static characteristic, *O_m_* and *O_m_^´^* are the balance points.

The arc self-regulating process in the GMAW welding process is shown in Figure 8. Where, *Mc* denotes the external characteristic of the welding machine. *S_c_* is the arc static characteristic curve and *S_c_^´^* represents the short arc static characteristic in the ultra-narrow groove gap. *W_m_* reflects that the wire melting rate equals to feeding speed. According to the minimum arc voltage principle, arc attachment point always selects the nearest conductive point to keep arc minimum energy consumption. With the groove gap decrease, the distance between wire tip and sidewall of groove decreases. Arc attachment point is on the sidewall of groove, the arc length becomes shorter, and the static characteristic curve is transformed from *S_c_* to *S_c_^´^*. At this point, the corresponding welding current increases from *I_m_* to *I_m_^´^*. The welding wire melting rate can be expressed by the following:

(7) $$v_{m}=\frac{1}{H_{0}+B}\left(rJ^{2}L+hJ\right)$$

where, *H_0_* is the enthalpy per unit volume, *B* is a constant, *r* is welding wire specific heat resistance, *J* is current density, *L* is wire extension length, and *h* is equivalent anode voltage drop. Formula 7 shows that the wire melting velocity *v_m_* is proportional to the square of welding current and the wire extension length.

The APCP process is shown in the Figure 8, it can be seen that welding current in balance point *O_m_^´^* is larger than that in the balance point *O_m_*. Hence, the corresponding wire melting velocity can be described by *v_m_^´^* > *v_m_*. Although the *O_m_^´^* meets the required characteristics of the welding machine and arc, it is hard to satisfy the balance between the welding wire melting rate *v_m_^´^* and wire feed speed *v_f_*. As mentioned above *v_m_^´^* > *v_m_*, so *v_m_^´^* > *v_f_*, hence the arc length will increase. However, when arc attachment point is on the sidewall, the distance between wire tip and sidewall is almost constant because of the fixed groove gap, the arc length does not change with the increase of wire melting velocity. The arc has to remain as melting wire to achieve a new balance point *v_m_^´^* = *v_f_*, which leads to a continuous decrease in the wire extension length. So the arc burning position moves up along the sidewalls as the wire extension length is reduced.

Furthermore, the smaller the groove gap is, the less the distance between wire tip and sidewall, and the farther the *O_m_^´^* goes from the balance point *O_m_*. This results in *v_m_^´^* > *v_f_*, and the speed of wire extension reduction becomes faster. Consequently, as the groove gap decreases, the speed of arc burning climbing up will be faster and the final arc burning position will be higher. Hence, the arc inherent self-regulation dose not adjust the arc length and arc burning position. So that in the ultra-narrow gap, the arc in GMAW welding process undergoes APCP and needs some extra methods to stabilize the arc process.

#### 3.3.2. Arc Burning Position in FBCA Welding

**Figure 9:**
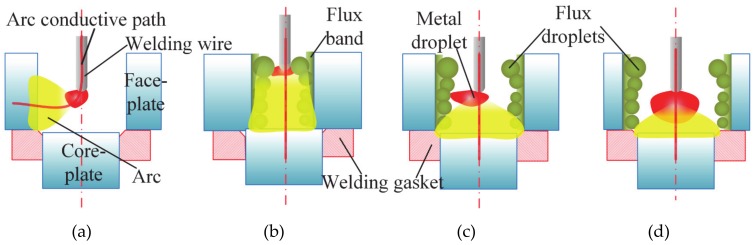
The arc behavior and droplet transfer modes in (**a**) GMAW and FBCA welding process under different groove gaps: (**b**) 3 mm (strong constricting effect) (**c**) 6 mm (medium constricting effect) (**d**) 10 mm (weak constricting effect).

After attaching the flux bands on the sidewalls in ultra-narrow gap groove, the arc burning positions in the FBCA welding process under different groove gaps are shown in Figure 9. It can be seen that the arc attachment point is fixed on the root of groove and the arc conductive path does not change with the variation of groove gaps. By comparing with the arc burning position in GMAW welding process, it indicates that the flux bands have a solid-wall constricting effect (SWCE) on the arc burning position because of the insulation of flux bands. The SWCE in ultra-narrow gap suppresses the arc column to conduct with nearest sidewall, eliminates the effect of the groove gaps on arc length, and effectively stabilizes the arc burning position.

The main process of SWCE adjusting the arc length and suppressing the APCP is analyzed as below: in the ultra-narrow gap FBCA welding process, when welding arc encounters interference and the arc length becomes shorter, the balance point *O_m_* moves to the *O_m_^´^*. At this moment, the welding current *I_m_^´^* > *I_m_*, the wire melting speed *v_m_^´^* > *v_m_*, so *v_m_^´^* > *v_f_*, the wire extension becomes shorter, and the arc length becomes longer, then the balance point is restored from *O_m_^´^* point to *O_m_*. Therefore, the SWCE keeps the arc length to follow the self-regulation process.

### 3.4. Effect of Flux Bands on Arc Shape

**Figure 10:**
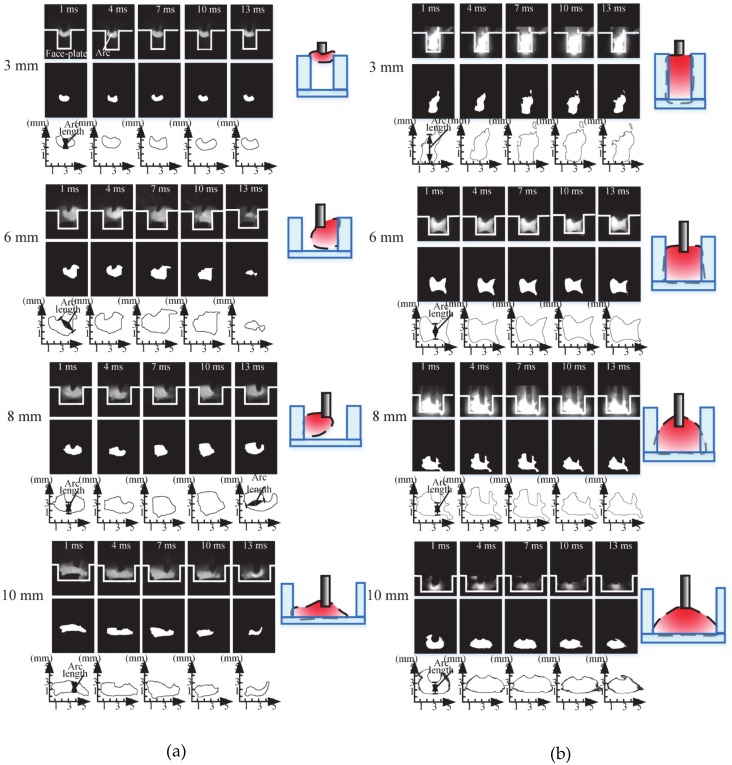
Simplified arc shape in the (**a**) GMAW and (**b**) FBCA welding process under the ultra-narrow groove gap.

In order to accurately extract the arc shape, the arc images in GMAW and FBCA welding process under different gaps are binarized, and the arc edge profiles are extracted to obtain simplified arc shapes, as shown in Figure 10. Figure 10a shows the arc shapes of GMAW welding in ultra-narrow gap groove without flux bands. It can be seen that the arc is in an unstable state without flux bands constraint, and the shape of the arc changes irregularly with the different conductive position. When the groove gap is 3 mm, the arc length is very short, and the arc shape is compressed into small spherical shape. When the groove gap is in the range of 6–8 mm, the arc burns on the side-wall, and the arc length increases slightly, presenting a small bell shape inclined to the side-wall. With the increase of gap, when the groove gap is larger than 10 mm, the influence of narrow gap side-wall on arc is weakened, and the shape of arc gradually returns to the shape of inverted bell. Figure 10b shows the appearance of the FBCA welding arc under the flux bands. It can be seen that the burning of FBCA welding arc is more stable than that of GMAW, and there is no irregular change in the arc shape. In the FBCA welding process, the arc is obviously squeezed by the flux bands, the arc width is equal to the groove gap, and the arc length increases obviously. When the groove gap is less than 5 mm, the arc almost fills the weld groove, showing a similar rectangular shape. With the increase of groove gap, the arc length gradually shortens. When the groove gap is 6–8 mm, the arc length is equal to the height of the groove, and the arc is square shape, which effectively heats the side-wall and the core-plate simultaneously. When the groove gap is larger than 10 mm, because the groove gap is larger than the arc width, the SWCE of flux bands on the arc is weakened, the arc length is further shortened, and the arc shape gradually returns to the inverted bell shape.

**Figure 11:**
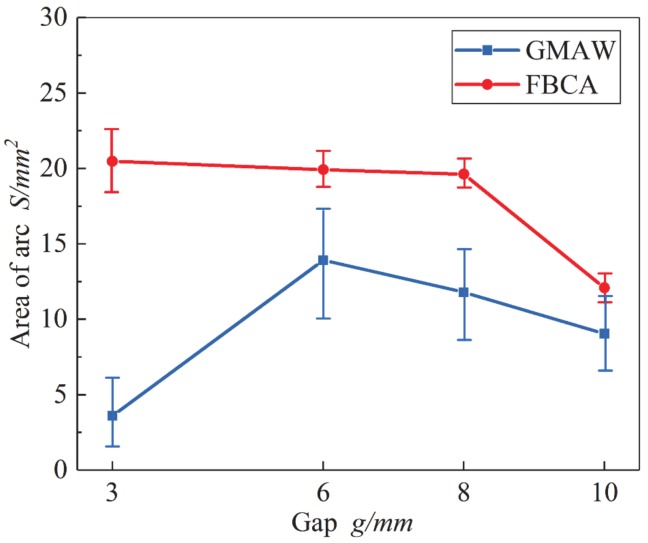
Influence of groove gaps on area of welding arc.

Based on the arc profiles images shown in Figure 10, the effective area of each arc profile image is calculated by pixel statistical method. Then the average values of effective area of welding arc under different groove gaps are calculated. The average arc area variation with groove gap is shown in Figure 11. It can be seen from the figure that in the ultra-narrow gap groove, when the weld groove gap *g* < 3 mm, the GMAW arc area is very small because of the limitation of the arc length. When the groove gap is more than 3 mm, all the arc area values as a whole become greater. However, with the increase of groove gap, the arc area decreases correspondingly. It shows that the groove also has a restraining effect on the arc, which is consistent with the conclusion of Meng [16]. In the process of FBCA welding, the effective area of welding arc is larger than that of GMAW. When the groove gap *g* < 3 mm, arc area does not decrease, but when the groove gap is 3–8 mm, the arc area is almost the same. When the groove gap *g* > 10 mm, the effective area of arc suddenly drops, and the arc area with or without flux bands constraint tends to be the same. This indicates that the flux bands are beneficial to the increase of the effective heating area of the arc. With the increase of the groove gap, the SWCE of the flux bands on arc weakens, so the arc area gradually returns to normal.

### 3.5. Effect of Flux Bands on Arc Heat

**Figure 12:**
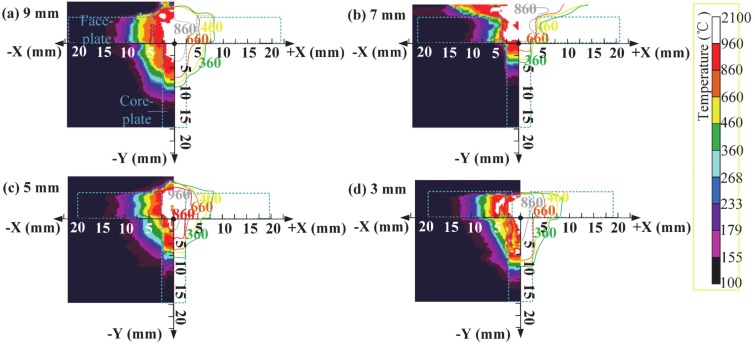
Temperature distribution of FBCA welding T-joint cross-sections under different groove gaps of (**a**) 9 mm, (**b**) 7 mm, (**c**) 5 mm, (**d**) 3 mm.

**Figure 13:**
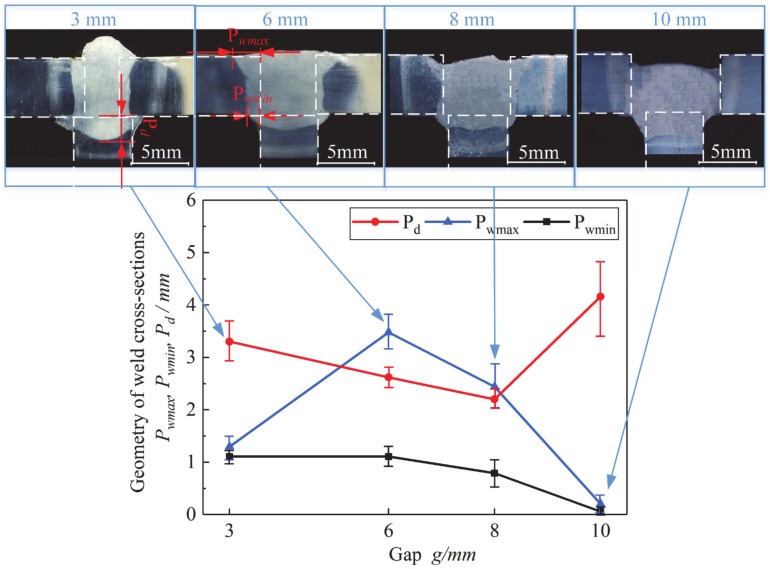
Weld cross-sections with different groove gaps.

In order to explore the effect of flux bands on the arc heat, the temperature distribution of the end-face of T-joint cross-section in FBCA welding process was measured by infrared temperature testing system. Figure 12 shows the temperature distribution of end-face of T-joint under different groove gaps during FBCA welding. Since the flux bands melt from around 600 °C, use the 660 °C isotherm as the analysis standard. It can be seen that the depth values of isotherm on the core-plate decrease from 8 mm to 3 mm with the increase of groove gaps from 3 mm to 7 mm. At the same time, the side-wall penetration of isotherm on the face-plate is about 5 mm. However, when the groove gap *g* ≥ 9 mm, the penetration of isotherm on the core-plate suddenly rises to 7 mm. Combined with the analysis of the T-joint cross-sections as shown in Figure 13, it can be seen that the ability of core-plate supporting the molten pool is reduced by the size limitation of the upper end-face of the core-plate. Weld leakage of core-plate will occur when the groove gap is larger than 9 mm. So the main reason for the increase of isotherm on the core-plate is that the overall heat source moves down due to the welding leakage of core-plate.

Because of the compressing effect of the flux bands on the arc, the temperature distribution of the arc heating the face-plates and the core-plate is changed, which significantly affects the weld formation including the side-wall and the core-plate penetration of the T-joint. Therefore, the analysis of the cross-section morphology under different groove gaps can further explain the effect of flux bands on the arc heat. The cross-sections of FBCA welding T-joint under different groove gaps are shown in Figure 13. It can be seen that the cross-section shape is almost same as the arc shape shown in Figure 10. The different arc shapes under different groove gaps determine the weld cross-section shapes. *P_D_* is defined as the penetration of core-plate, *P_wmax_* as the maximum penetration of side-wall, and *P_wmin_* as the minimum penetration of side-wall. Except that the heat source moves down when the core-plate has a weld leakage at 10 mm, the penetration of core-plate increases with the decrease of groove gap when groove gap is in the range of 3–8 mm. It indicates that the flux bands not only compress the arc shape, but also intensifies the heating degree of the arc to the core-plate. At the same time, the *P_wmin_* is basically the same, while the *P_wmax_* increases first and then decreases. It shows that flux bands regulate the heat of arc heating side-wall. A sound flux bands melting state obtains a good side-wall penetration. Under strong constricting effect, the maximum penetration of side-wall is insufficient, while weak constricting effect is not conducive to side-wall fusion. Meanwhile when groove gap is larger than 8 mm, the weld is not fulfilled. The main reasons are: 1. With the groove gap increase, the amount of melting wire is not enough to fill up the groove, 2. the probability of welding leakage of core-plate increases with the increase of groove gap.

### 3.6. Effect of Flux Bands on Metal Transfer

**Figure 14:**
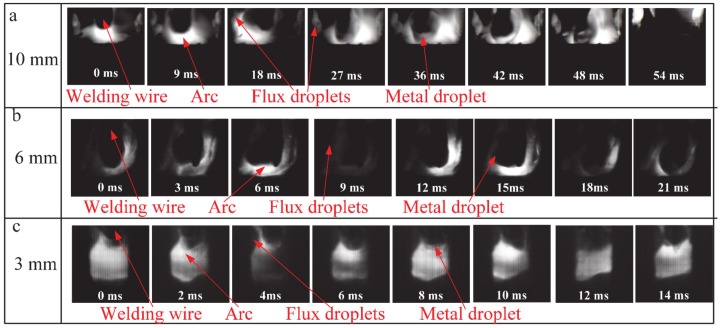
The metal transfers under different groove gaps in FBCA welding process. (**a**) 10 mm, (**b**) 6 mm, (**c**) 3 mm.

The metal transfer characteristics are the important factors that significantly affect the arc behavior and welding process. The metal transfer characteristics in FBCA welding under different groove gaps are also different from that in traditional GMAW, as shown in Figure 14. It can be seen that there are flux droplets in the FBCA welding process, and the interaction process between the flux droplets and metal droplet determines the unique metal transfer characteristics of FBCA welding. When the groove gap is 10 mm, the flux droplets hardly contact with the metal droplet because of the insufficient heating of arc on flux bands. The wide groove gap results in the metal droplet has enough space to grow up. The metal droplet continuously grows until contacts the molten pool of core-plate; it shows a typical short-circuiting transfer feature. When the groove gap is 3 mm, because of space limitation, there is almost no space for metal droplet to grow, the flux droplets constantly contact the metal droplet. The metal transfer is flux-wall guided transfer. When the groove gap is 6 mm, the metal droplet not only contacts with the flux droplets, but also with the molten pool with the growth of the metal droplet. The metal transfer characteristic is a mixed transfer mode. The above analysis further shows that the probability of flux-wall guided transfer will increase with the decrease of groove gap.

### 3.7. Effect of Flux Bands on Arc Stability

**Figure 15:**
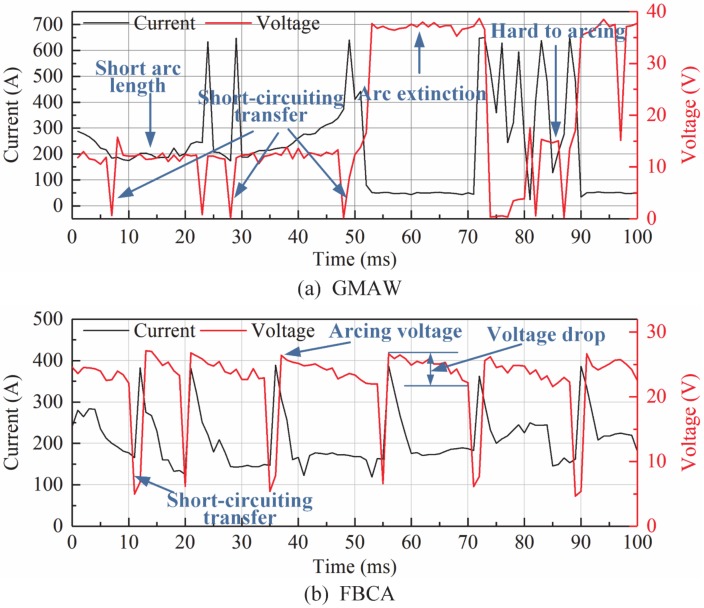
The dynamic characteristics of voltage and current in GMAW and FBCA welding process (welding current: 260 A, arc voltage: 24 V, welding speed: 7.5 mm/s, groove gap: 6 mm). (**a**) GMAW, (**b**) FBCA.

The instantaneous values of welding current and arc voltage in welding process are collected within randomly selected 0.1 s period. The dynamic characteristics of arc voltage and welding current before and after adjusting flux bands (GMAW and FBCA) are drawn by the instantaneous values as shown in Figure 15. It can be seen that the arc voltage and welding current fluctuate greatly, the arc is difficult to arcing, and there is a long arc extinction period in GMAW process. After adding flux bands, the FBCA welding process presents a typical short-circuiting transfer waveform. The short-circuiting transfer frequency is about 17.8 ms, and the welding process is relatively more stable than GMAW.

**Figure 16:**
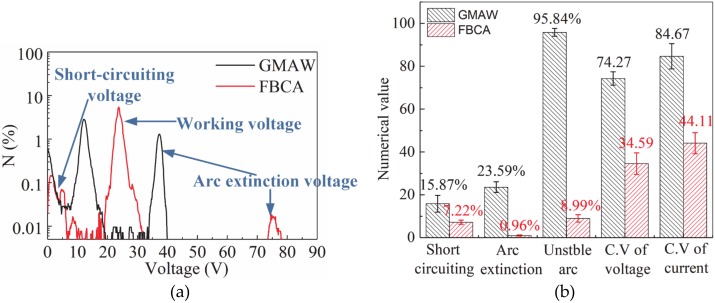
(**a**) Probability distribution of arc voltage and (**b**) the analysis of arc stability in GMAW and FBCA welding process.

The probability (*N*%) distribution of voltage diagram reflects the distribution of arc voltage values in the whole welding process [28], which can be drawn by the following steps: First count the number of arc voltage values in every 0.05 V incremental intervals, then calculate the proportion of each interval, and finally draw the curves of the proportion of each interval variation with the median of interval, as shown in Figure 16a. The probability distribution of voltage shows that in the ultra-narrow gap groove, there are two “humps” (13 V and 37 V) and there are no normal working voltage “humps” (24 V) in GMAW welding, indicating that the arc length is changeful. The arc conductive path wanders between the side-wall and the core-plate. On the contrary, there is a higher normal working voltage “Hump” (24 V) and lower short-circuiting voltage and arc extinction voltage “humps” in the FBCA welding process, which show that the arc stability is significantly improved by flux bands. 

A subsequent quantitative analysis about the average values of proportion of arc extinction time, the proportion of short-circuiting time, the proportion of unstable arc burning time, and the coefficient of variation (*C∙V*) of arc voltage and welding current are performed as shown in Table 3. The average values are calculated by the corresponding results of three groups of repeated GMAW and FBCA welding experiments under similar parameters. Set the 15 V as the short-circuiting voltage threshold and 35 V as the arc extinction voltage threshold. The time of arc voltage in the range of 0–15 V and >35 V is calculated as unstable arc burning time. The coefficient of variation (*C∙V*) is shown in Equation (8), which can evaluate the fluctuation degree of values.

(8) $$C\cdot V=\frac{\sigma }{\mu }$$

(9) $$\sigma =\sqrt{\frac{1}{N}\sum_{i=1}^{N}{\left(x_{i}-\mu \right)}^{2}}$$

where *σ* is the standard deviation and *μ* is the average of a set of values. The analysis of arc stability is shown in Figure 16b, it can be seen that the proportion of short-circuiting time, arc extinction time, and unstable arc burning time are reduced by 8.65%, 22.63%, and 86.85%. The fluctuation of voltage and current are also significantly reduced by the flux bands. Consequently, the flux bands effectively increase the arc stability in the ultra-narrow gap through its SWCE on the arc.

## 4. Conclusions

First, this paper introduces the state of flux bands at high temperature, and presents the mechanism of constricting effect of flux bands on arc by comparing the position of arc burning, arc shape, arc heat, and arc stability of GMAW and that of FBCA welding under different weld groove gaps. The specific conclusions are as follows:

(1) Flux bands localized melting start at 600 °C, then change from solid state to gas-liquid mixture state at 800 °C. Non-conductive flux bands will have a solid-wall constricting effect (SWCE) on the arc. The flux bands decompose in high temperature to product CO_2_ gas and CaO-CaF_2_ slag system, which can protect the welding process with the function of self-producing slag and gas. The flux bands also have a thermo-compression effect (TCE) on the arc as the melting of flux bands takes away part of the heat of the arc.
(2) In ultra-narrow groove gap, arc in GMAW welding process undergoes APCP, the SWCE of flux bands on arc suppresses the APCP because of the insulation of flux bands.
(3) Arc shapes of GMAW welding in ultra-narrow gap without flux bands changes irregularly with the different conductive position, and arc burning state is unstable. FBCA welding arc is more stable than GMAW. In the FBCA welding process, the arc is obviously squeezed by flux bands from an inverted bell shape to a rectangle shape, the arc width is equal to the groove gap, and the arc length increases obviously. The effective heating area of the arc is increased by at least 5 mm^2^ compared with that in GMAW, which indicates that the flux bands are beneficial to the increase of the effective heating area of the arc.
(4) The temperature distribution of the end-face of T-joint cross-section in FBCA welding process shows the 660 °C isotherm on the core-plate increase from 3 mm to 8 mm with the groove gap increasing from 7 mm to 3 mm. The FBCA welding T-joint cross-sections show that flux bands regulate the heat of arc heating side-wall: Under strong constricting effect (groove gap is 3 mm), the maximum penetration of side-wall is 1.3 mm, while the maximum penetration of side-wall increases to 3.5 mm under medium constricting effect (groove gap is 6 mm). The maximum and minimum penetration of side-wall decrease to 0 mm when groove gap is 10 mm, indicating that weak constricting effect is not conducive to side-wall fusion.
(5) The proportion of short-circuiting time, arc extinction time, and unstable arc burning time are respectively reduced by 8.65%, 22.63%, and 86.85%, and the fluctuation of voltage and current are significantly reduced by the flux bands. The flux bands effectively increase the arc stability in the ultra-narrow gap through its SWCE on the arc.

## Figures and Tables

**Table 1:** Welding parameters.

Case No.	Arc Voltage (U/V)	Welding Current (I/A)	Welding Velocity (v/mm·s^−1^)	Groove Gap (g/mm)
Case 1	21, 24, 30	260	7.5	6
Case 2	24	180, 260, 300	7.5	6
Case 3	24	260	7.5	3, 6, 8, 10

**Table 2:** The chemical compositions of flux band (wt. %).

Marble	Fluorite	Titanium Dioxide	Mica	Ferrotitanium	Sodium Silicate	Others
40	27	7	2	15	1	8

**Table 3:** Analysis results of arc stability.

Experiments No.	Proportion of Short-Circuiting Time (%)	Proportion of Arc Extinction Time (%)	Proportion of Unstable Arc Burning Time (%)	C·V of Voltage	C·V of Current
1	19.99	26.40	96.34	77.65	90.17
2	12.17	21.68	93.82	71.45	78.46
3	15.46	22.72	97.35	73.71	85.40
*GMAW average*	*15.87 ± 3.93*	*23.59 ± 2.48*	*95.84 ± 1.82*	*74.27 ± 3.14*	*84.67 ± 5.89*
1	8.19	1.28	9.78	40.37	48.62
2	6.21	0.77	6.99	31.25	38.88
3	7.27	0.82	10.20	32.16	44.83
*FBCA average*	*7.22 ± 0.99*	*0.96 ± 0.28*	*8.99 ± 1.75*	*34.59 ± 5.02*	*44.11 ± 4.91*
